# Electrochemical and biological performance of hierarchical platinum-iridium electrodes structured by a femtosecond laser

**DOI:** 10.1038/s41378-022-00433-8

**Published:** 2022-09-02

**Authors:** Linze Li, Changqing Jiang, Wanru Duan, Zhiyan Wang, Feng Zhang, Changgeng He, Tiangang Long, Luming Li

**Affiliations:** 1 National Engineering Research Center of Neuromodulation, School of Aerospace Engineering, Tsinghua University, Beijing, 100084 China; 2 School of Mechanical Engineering and Automation, Fuzhou University, Fuzhou, 350108 China; 3 Department of Neurosurgery, China International Neuroscience Institute (CHINA-INI), Xuanwu Hospital, Capital Medical University, Beijing, 100053 China; 4 Lab of Spinal Cord Injury and Functional Reconstruction, China International Neuroscience Institute (CHINA-INI), Xuanwu Hospital, Capital Medical University, Beijing, 100053 China; 5 Precision Medicine & Healthcare Research Center, Tsinghua-Berkeley Shenzhen Institute, Tsinghua University, Shenzhen, 518071 China; 6 IDG/McGovern Institute for Brain Research at Tsinghua University, Beijing, 100084 China; 7 Institute of Epilepsy, Beijing Institute for Brain Disorders, Beijing, 100093 China

**Keywords:** Nanostructures, Electrical and electronic engineering

## Abstract

Neural electrode interfaces are essential to the stimulation safety and recording quality of various bioelectronic therapies. The recently proposed hierarchical platinum-iridium (Pt-Ir) electrodes produced by femtosecond lasers have exhibited superior electrochemical performance in vitro, but their in vivo performance is still unclear. In this study, we explored the electrochemical performance, biological response, and tissue adhesion of hierarchical Pt-Ir electrodes by implantation in adult rat brains for 1, 8, and 16 weeks. Regular smooth Pt-Ir electrodes were used as a control. The results showed that the electrochemical performance of both electrodes decreased and leveled off during implantation. However, after 16 weeks, the charge storage capacity of hierarchical electrodes stabilized at ~16.8 mC/cm^2^, which was 15 times that of the smooth control electrodes (1.1 mC/cm^2^). Moreover, the highly structured electrodes had lower impedance amplitude and cutoff frequency values. The similar histological response to smooth electrodes indicated good biocompatibility of the hierarchically structured Pt-Ir electrodes. Given their superior in vivo performance, the femtosecond laser-treated Pt-Ir electrode showed great potential for neuromodulation applications.

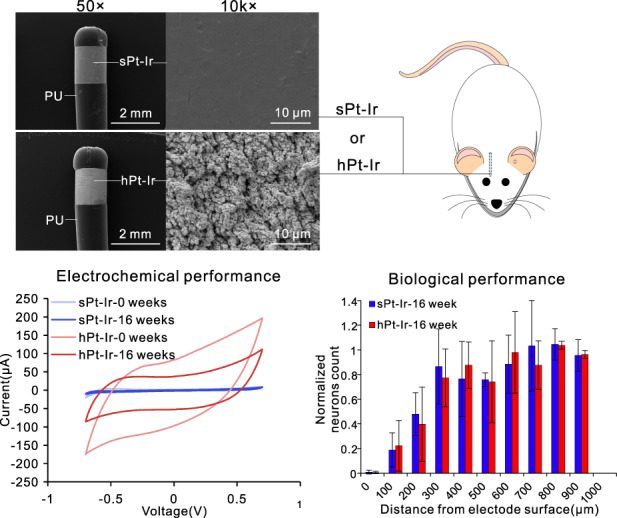

## Introduction

Neural implants have been remarkably successful in treating various neurological and psychiatric disorders and have shown great potential for brain-computer interfaces (BCIs)^1^. As an important component of both neuromodulation and BCI devices, the implanted electrodes for stimulation and/or recording must meet the requirements of safe charge transfer and minimal impedance changes^2^.

Platinum-iridium alloy (Pt-Ir), with its excellent biocompatibility and corrosion resistance, has been widely used in implanted neural electrodes, such as deep brain stimulation (DBS), stereotactic electroencephalography, and cerebral cortex electrodes^3^. To achieve more precise stimulation and recording with higher spatial resolution, electrodes are becoming smaller^4^. The reduced electrode size leads to increased stimulation charge density, which tends to exceed the electrochemical limits of Pt-Ir electrodes^2,5^. In addition, smaller electrodes are usually accompanied by a high impedance, which not only reduces stimulation efficiency but also affects recording quality^1^.

Various methods have been reported to improve the charge transfer and reduce the electrochemical impedance of Pt-Ir electrodes, typically including coating^6–9^ and surface modification^10,11^. These approaches can significantly enhance the charge storage capacity and reduce the electrode impedance; however, there are also challenges limiting their practical applications, such as biotoxicity and lack of long-term stability of materials^1^.

Recently, femtosecond laser direct writing, another surface modification technique, has emerged as one of the best methods for creating micro/nanostructures on metal surfaces due to its flexibility, simplicity, and controllability^12,13^. Through processes such as reconsolidation after ablation and redeposition of nanoparticles, various micro/nanostructures can be produced on Pt-Ir surfaces^13^. After optimization of the processing strategy, the in vitro charge storage capacity of hierarchical Pt-Ir electrodes has been shown to increase by more than an order of magnitude with highly reduced interface impedance^14^.

For long-term applications, neural electrodes must have good electrochemical properties and biocompatibility in vivo^1^. Although Pt-Ir electrodes have demonstrated stable performance for decades in clinical use, the new surface structures may have an impact on cell and tissue adhesion, thus affecting their performance^15^. Earlier, an in vivo study showed that there were different biological adhesions and changes in electrochemical performance for smooth and porous titanium nitride electrodes after several weeks of implantation in the rat dorsum^16^. However, the foreign body response could be influenced by the implanted regions and surface structures^15^. The electrochemical properties and long-term biocompatibility of hierarchical Pt-Ir neural electrodes within the brain remain unclear.

In this study, both hierarchical and smooth Pt-Ir electrodes were implanted in adult rat brains to investigate the effects of the surface structures on the electrochemical performance and electrode-tissue interface. The in vivo acute and chronic studies were expected to assess the clinical feasibility of the novel electrode architecture.

## Materials and methods

### Experimental design

**Fig. 1:**
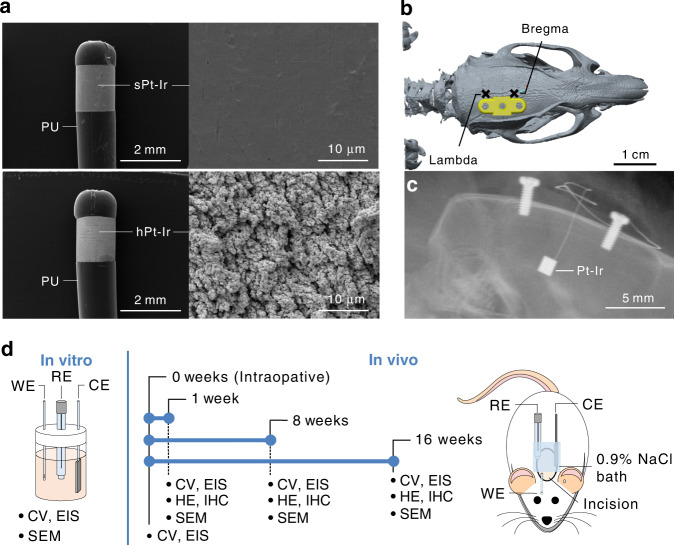
Study overview. **a** Smooth and hierarchical Pt-Ir electrode surfaces (sPt-Ir and hPt-Ir). PU polyurethane. **b** Fixation base of the electrode^17^. **c** X-ray photo of the implanted electrode. **d** Experimental design. WE working electrode, RE reference electrode, CE counter electrode, CV cyclic voltammetry, EIS electrochemical impedance spectrum, HE hematoxylin–eosin staining, IHC immunohistochemistry, SEM scanning electron microscopy.

The study overview and experimental schedule are illustrated in Fig. 1. Sprague–Dawley rats aged 6~8 weeks were randomly assigned to the smooth Pt-Ir (sPt-Ir) and hierarchical Pt-Ir (hPt-Ir) groups (Table 1). In each group, 18 rats were equally distributed to 3 implantation durations: 1, 8, and 16 weeks. Each rat was implanted with 1 electrode. Electrochemical testing and surface characterization were performed before and after implantation, and histological sections were analyzed at every implantation endpoint. The procedure was approved by the Institutional Animal Care and Use Committee of Tsinghua University and followed the requirements for animal care and handling.

**Table 1:** Groups of experimental animals.

Electrode type	Animal number		
	1 week	8 weeks	16 weeks
sPt-Ir	6	6	6
hPt-Ir	6	6	6

### Electrode fabrication

Bare Pt-Ir alloy tubes with an outer diameter of 1.27 mm and a wall thickness of 100 μm were used (90% Pt and 10% Ir, Johnson Matthey, USA). Hierarchical structures on Pt-Ir were produced by a previously described femtosecond laser system with the following optimized parameters: pulse energy of 10 μJ, the repetition rate of 100 kHz, scanning speed of 5 mm/s, and scanning interval of 8 μm^14^. After laser treatment, the Pt-Ir tube was cut off with a length of 1.5 mm to form electrode contacts. Then, debris was removed from the surface by ultrasonic cleaning in water for 30 s (Fig. 1a). The sPt-Ir was directly cut from the Pt-Ir tube without laser treatment. The contacts were connected to 0.1 mm diameter MP35N wires (Fort Wayne, USA) and set on a 1.3 mm outer diameter polyurethane tube (Lubrizol, USA). The electrodes were fixed on a photosensitive resin mounting base (DSM, Netherlands) conforming to the rat skull (Fig. 1b)^17^.

### Surgical procedure

Each animal was anesthetized with 2% sodium pentobarbital (40 mg/kg) and then fixed by the ear bars of a stereotaxic frame (World Precision Instrument, USA). After shaving the scalp, the skin was incised to expose the bregma. The electrodes were implanted toward the right subthalamic nucleus, but a shallower position was chosen to avoid stabbing through the brain. The coordinates were determined according to George Paxinos & Charles Watson’s stereotaxic atlas of the rat brain^18^: 3.8 mm behind the bregma, 2.5 mm to the midline, and 6 mm subdurally. A randomly selected sPt-Ir or hPt-Ir electrode was implanted and fixed by stainless steel screws (Fig. 1c).

### Behavioral and health assessments

Overall survival and postoperative behavior were recorded for all animals. Weekly body weight was assessed as an indicator of general health.

### Electrochemical testing

Electrochemical performance was tested using an electrochemical workstation (CHI 660E, CHI, China). In vitro tests were performed in phosphate-buffered saline (0.01 M, Solarbio, China). In vivo tests were conducted during surgery and before sacrifice with a physiological saline bath on the skull formed by medical skimmed cotton. A three-electrode system was used for both in vitro and in vivo testing (Fig. 1d): an sPt-Ir or hPt-Ir electrode as the working electrode, a titanium sheet (20 mm × 30 mm × 0.3 mm) as the counter electrode, and an Ag/AgCl electrode filled with saturated KCl (Model 218, Leici, China) as the reference electrode. During the in vivo test, a ball of medical skimmed cotton soaked with physiological saline was first placed on the skull to form a bath, and then the counter electrode and reference electrode were immersed into it. After testing, they were removed.

First, the cyclic voltammetry (CV) scans started from the open circuit potential in a voltage range from −0.7 V to 0.7 V versus the reference electrode at a scan rate of 50 mV/s. The charge storage capacity (CSC) was obtained by integrating the cathode current density over time. After the CV scans, the electrochemical impedance spectrum (EIS) was measured using the same instruments. A sinusoidal voltage excitation of 10 mV was used, and the test frequency range was from 0.1 Hz to 100 kHz. The cutoff frequency was marked in the EIS corresponding to the frequency at the phase of −45°, which indicated the high and low impedance amplitude boundaries^19^.

### Histological characterization

After electrochemical testing at 1, 8, and 16 weeks, the animals were euthanized with an overdose of sodium pentobarbital and systemically perfused with phosphate buffer and 4% formaldehyde solution. Then, the electrodes were gently explanted, and the brain tissues were carefully removed. The brain tissues and electrodes were fixed separately in 4% formaldehyde for 24 h. The brain tissue was embedded in paraffin wax. Perpendicular to the electrode axis, consecutive sections were made at a thickness of 5 μm. After dewaxing with xylene, some sections were stained with hematoxylin–eosin (HE). The other sections were incubated with antibodies: rabbit anti-neuronal nuclei (NeuN, AB177487, Abcam, UK, 1:1000), mouse anti-glial fibrillary acidic protein (GFAP, MAB360, Millipore, USA, 1:800) and rabbit anti-ionized calcium binding adaptor molecule 1 (Iba-1, AB178846, Abcam, UK, 1:2000) at 4 °C overnight to label neurons, astrocytes, and microglia, respectively. Then, appropriate dilutions of horseradish peroxidase-conjugated secondary antibodies were added, followed by 3,3′-diaminobenzidine (DAB, PV-9001 and PV-9005, ZSGB-Bio, China).

Photographs were taken at ×20 magnification using an automated digital slide scanner (Axio Scan.Z1, Zeiss, Germany). The measurements were conducted on one NeuN-stained slide for each animal. The normalized neuron number was calculated as a function of distance from the electrode-tissue interface using the image-processing program ImageJ (National Institute of Health)^20–22^. An outline of the electrode-tissue interface was defined automatically. NeuN-positive cells were counted at 100 μm increment bins away from the interface up to 1000 μm using a marker-controlled watershed algorithm in ImageJ. The results were normalized to the background cellular count (1000 μm away from the interface). The average values and standard deviations were calculated.

### Scanning electron microscopy

The explanted electrodes were dehydrated using graded ethanol and tert-butanol and then lyophilized at −20 °C. The electrodes were observed using a scanning electron microscope (SEM) operating at 20 kV (Quanta 200, FEI, Netherlands). Each electrode was photographed at magnifications of ×100, ×1k, and ×10k to examine the surface structure and biological adhesion.

### Statistical analysis

Data were processed by SPSS (Version 24, IBM, USA). Differences were analyzed using one-way analysis of variance (ANOVA) and *t* tests. *P* values < 0.05 were reported as statistically significant.

## Results

### Behavioral and health assessments

**Fig. 2:**
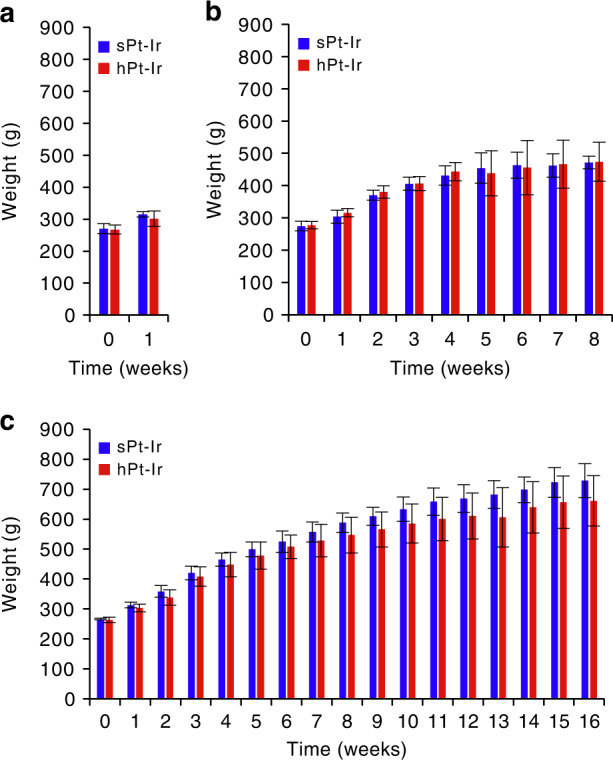
Weekly body weight. Rats implanted for **a** 1 week, **b** 8 weeks and **c** 16 weeks.

All animals survived to the scheduled implantation time. No behavioral abnormalities indicative of central nervous system damage, such as tremor or slow or skewed movement, were observed. The weekly body weight of the rats gradually increased with time over the 3 implantation durations. While the mean endpoint weight of the hPt-Ir groups was larger at 8 weeks and smaller at 1 and 16 weeks of implantation than that of the sPt-Ir groups, there was no significant difference between the two electrode groups (*p* > 0.05), as shown in Fig. 2.

### Electrochemical performance

**Fig. 3:**
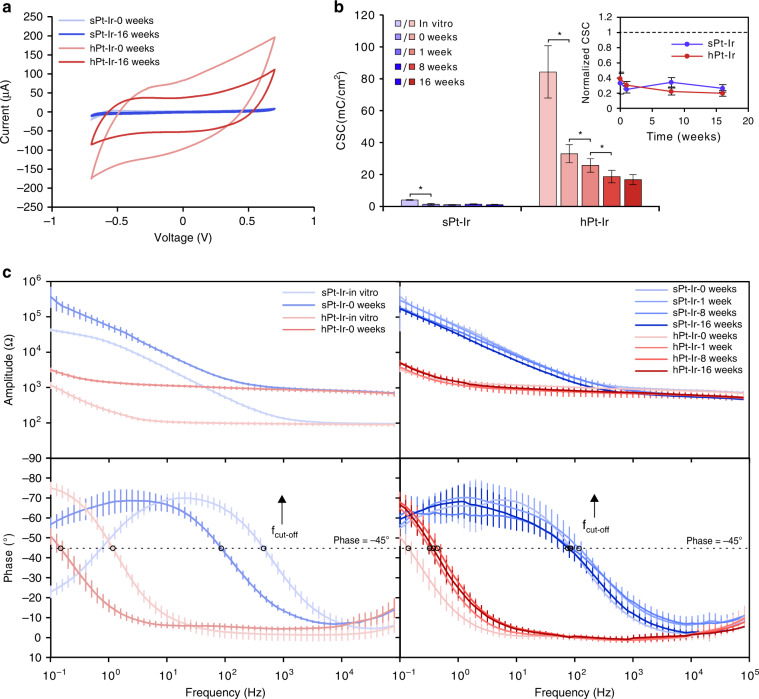
Electrochemical performance results. **a** Cyclic voltammetry curves of sPt-Ir and hPt-Ir at 0 and 16 weeks. **b** In vitro and in vivo charge storage capacity (CSC). The inset shows the trend of normalized CSC varying with implantation time compared to the in vitro value. **c** In vitro and in vivo electrochemical impedance spectra. An asterisk (*) denotes *p* < 0.05 (one-way ANOVA).

The CSC results obtained by CV are shown in Fig. 3a, b. Compared with the preimplantation levels in vitro, the intraoperative CSCs (at 0 weeks) of sPt-Ir and hPt-Ir decreased by 66.4% ± 15.5% and 60.2% ± 6.8%, respectively (*p* > 0.05), which showed the effects of the in vitro and in vivo environments. With a longer implantation time, the CSCs of sPt-Ir electrodes exhibited no significant change, but the CSCs of hPt-Ir electrodes further decreased and then tended to be stable after 8 weeks of implantation. At 16 weeks, while the CSC of the hPt-Ir electrode (16.8 mC/cm^2^) decreased to 47.7% of the intraoperative level, it was still 15 times that of the smooth control electrode (1.1 mC/cm^2^).

The changes in EIS are shown in Fig. 3c. Compared with that in the in vitro environment, an increase in impedance amplitude and a decrease in cutoff frequency were observed for both electrodes after implantation at 0 weeks. During the implantation time from 0 to 16 weeks, the EIS amplitude showed smaller changes than during surgery. In addition, the cutoff frequency tended to be stable for sPt-Ir but increased for hPt-Ir in vivo. After 16 weeks of implantation, the impedance amplitude and cutoff frequency of the hPt-Ir electrode remained significantly lower than those of sPt-Ir. The low impedance amplitude and cutoff frequency could help to reduce stimulation power consumption and record weak electrophysiological signals.

### Histological analysis

**Fig. 4:**
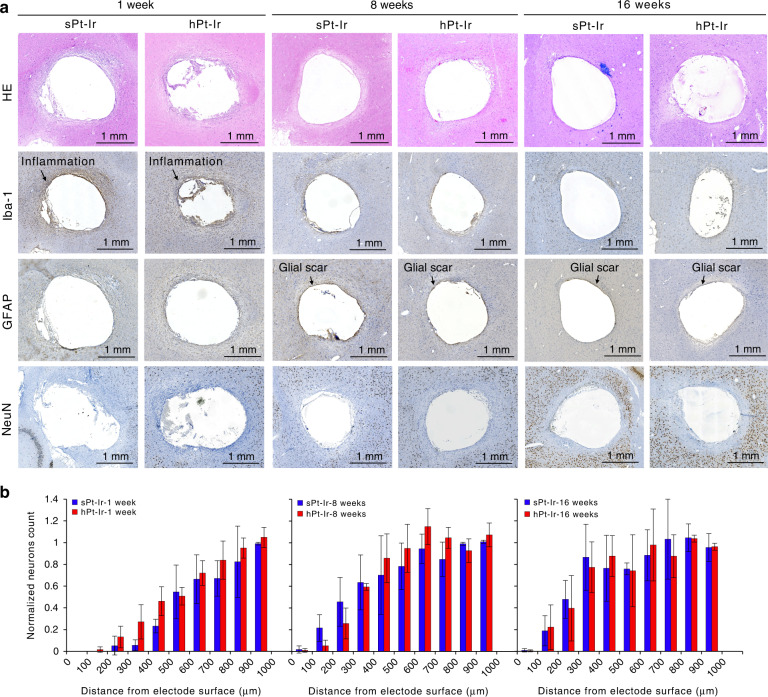
Histological results. **a** HE, Iba-1, GFAP, and NeuN staining of the tissue surrounding the electrodes at 1, 8, and 16 weeks after implantation. **b** Normalized neurons count with increasing distance from the electrode interface.

HE and immunohistochemistry staining are shown in Fig. 4a). In HE staining, a distinctive increase in the number of cells within a few hundred micrometers around both the sPt-Ir and hPt-Ir electrodes could be observed at 1 week, indicating an acute inflammatory response. This was verified by the denser microglia (Iba-1 staining) surrounding the electrodes. By 16 weeks, the dense microglia were no longer evident in either group, suggesting gradual healing of the implantation wound. In contrast to the predominance of inflammation at 1 week, the GFAP-stained glial scar became apparent at 8 weeks postimplantation and became thinner at 16 weeks. Correspondingly, the NeuN-stained neuron-depleted area became narrow, and the neuronal density within 500 μm of both electrodes consistently increased during implantation (Fig. 4b). No significant difference was found between the two groups (*p* > 0.05). In all stained sections, no shedding of particulate material was observed around the electrodes.

### Electrode surface analysis

**Fig. 5:**
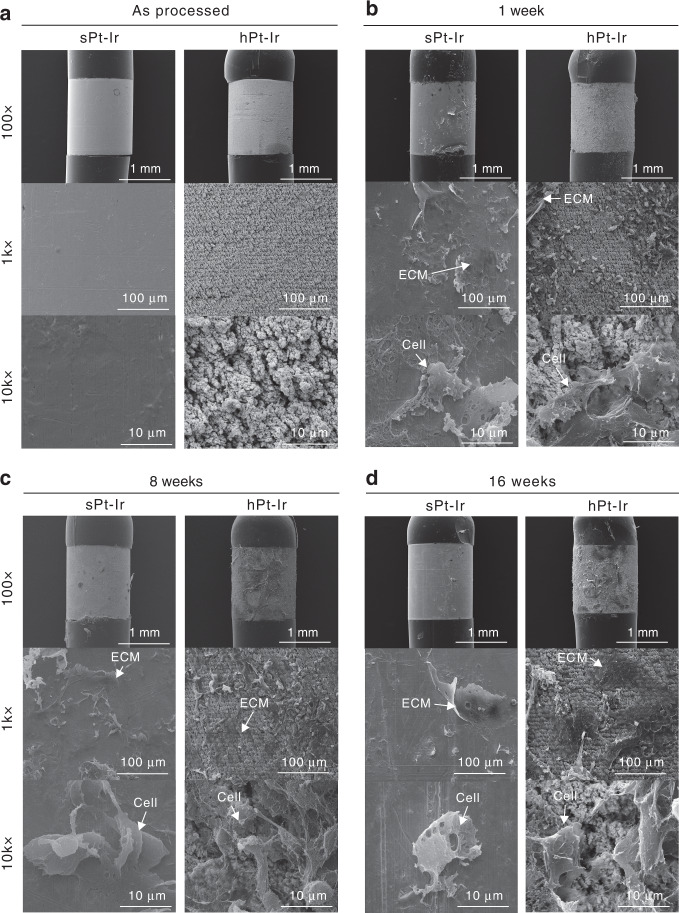
Scanning electron microscopy of electrodes surfaces. The sPt-Ir and hPt-Ir electrodes surfaces **a** as processed and after **b** 1 week, **c** 8 weeks, and **d** 16 weeks of implantation.

SEM characterizations of the explanted electrodes are shown in Fig. 5. While biological adhesion was not evident on the sPt-Ir and hPt-Ir surfaces at ×100 magnification, extracellular matrix (ECM) and cells could be found on both electrodes at higher magnification (×1k and ×10k). For a longer implantation time, there was no distinctive change in biological adhesion on sPt-Ir, whereas more tissue adhesion could be found on hPt-Ir electrodes, especially at 8 and 16 weeks. This may be associated with the decreased CSC of hPt-Ir electrodes after long-term implantation (Fig. 3b).

## Discussion

Electrochemical performance and biocompatibility are critical for neural electrodes. To assess clinical feasibility, hierarchical Pt-Ir electrodes structured by a femtosecond laser were implanted in rat brains to study their in vivo acute and chronic performance compared with existing smooth Pt-Ir electrodes^23^. Although both electrodes experienced a decrease in charge storage capacity after implantation, the hierarchical Pt-Ir electrode showed superior long-term electrochemical performance and comparable biocompatibility to the smooth electrode, demonstrating its potential for application as a clinical neural electrode.

### Influence of implantation on electrochemical performance

Compared with the in vitro results (Fig. 3b), the intraoperative CSCs for both the sPt-Ir and hPt-Ir electrodes decreased at a similar rate. This decrease is reported to be associated with the reduced ionic conductivity and oxygen concentration of the biological environment^24^. Over the course of the experiment, the CSCs of the sPt-Ir electrodes remained stable, but the values of the hPt-Ir electrodes further decreased by ~50% from 0 to 8 weeks (Fig. 3b), similar to that of the electrodeposited Pt-Ir coating on cochlear electrodes^25^. Given that the hierarchical structures showed no signs of shedding, the deterioration should be related to the glial scarring and biological adhesion on the electrode surface, as seen in the histology and SEM characterizations (Figs. 4 and 5)^26–28^. In the surrounding blood and extracellular matrix, serum albumin and procollagen are the most common proteins, respectively, both with diameters of a few nanometers^16^. These proteins might enter the submicron-sized pores of the hPt-Ir electrode surface, leading to an increase in the pore resistance and limiting the inward diffusion of ions^16,29,30^. With complete encapsulation, the electrochemical performance of hPt-Ir tended to be stable after 8 weeks^31^.

The differences in EIS among various implantation times were smaller than those between in vitro and intraoperative measurements (Fig. 3c). This was consistent with previous studies^16,32^. Not only in vitro but also in vivo, the hPt-Ir electrodes had a similar impedance above the cutoff frequency compared to sPt-Ir electrodes. The impedance in this range was nearly frequency-independent and mainly determined by the geometric surface area and the surrounding electrolytes^19^. On the other hand, below the cutoff frequency, the hPt-Ir electrodes had a lower impedance. The impedance in this range was frequency-dependent and related to the real surface area^19^. In addition, the postoperative f_cut-off_ of the hPt-Ir electrode was slightly higher than the intraoperative f_cut-off_ reflected by the rightward shift of the phase curve. This result suggested an increased real part of the impedance, which might result from increased biological adhesion^31^. Nevertheless, after 16 weeks of implantation in the brain, the hPt-Ir electrode still had greater strengths in impedance amplitude and cutoff frequency.

Intriguingly, the hPt-Ir electrode had a rather stable EIS over 16 weeks, but the CSC value decreased significantly over the first 8 weeks. Similar results have also been found in conductive hydrogel electrodes in rats^22,33^. It is assumed to be associated with varying diffusion processes under CV and EIS tests. Given the complex effects of electrode materials, surface structures, and implantation environments on electrochemical processes, it would be worthwhile to conduct more research in the future.

### Biocompatibility of the structured electrodes

Biocompatibility is another key aspect of neural implants, which is characterized by increased surrounding neuron density and limited glial encapsulation and inflammation^34,35^. Smooth Pt-Ir electrodes have been widely used in long-term implants and have shown good biocompatibility^19^. Nevertheless, according to ISO 10993-22: 2017^36,37^, medical devices containing nanostructured surfaces should prevent nanoparticle release and should be assessed for their potential effects on cells and tissues due to direct interactions.

To investigate the effects during acute and long-term implantation, we examined the progression from 1 to 16 weeks^21,34,35^. In histological analysis, there were similar neuronal densities and thicknesses of neuron-depleted areas around both electrodes, indicating no distinctly negative impact of hPt-Ir (Fig. 4). Furthermore, previous studies have shown that activated microglia and an inflammatory response would be observed if implants were causing the shedding and spreading of micro/nanoparticles^38,39^. In this study, no particulate material from micro/nanostructures or abnormal inflammation was observed in the tissues, indicating that the hierarchically structured Pt-Ir electrodes had good mechanical stability and biocompatibility.

The neuron distribution around the electrodes is related to the biocompatibility and functionality of the electrodes. After 16 weeks of implantation, there were fewer neurons within 200~500 µm of both electrodes than there were farther away from the electrodes. A similar neuronal distribution has been found in previous studies^21,22^. Neuronal migration is an important issue for microelectrodes due to their limited accessible tissue volume. However, this migration has less of an effect on the macroelectrodes used in this study. These electrode sizes can be used to stimulate neurons and axons^40^ and record local field potentials^41^ within a few millimeters. Although the electrodes reported here are functional, improvements in biocompatibility are of great value and deserve further investigation.

Using nanostructured surfaces to control protein and cell adhesion on electrodes has also attracted increasing attention^15,42,43^. Several in vitro studies have shown that nanostructures may preferentially provide architectural cues for neuronal adhesion compared to astrocytes^44,45^. In addition, structured microelectrodes have been reported to enhance glial resistance^46^ and reduce the expression of some inflammation-related genes after implantation within the brain^47^. These results illuminate the improved integration between neurons and electrodes, which enhances stimulation efficiency and recording quality^34,43,44,48^. In this study, hierarchical Pt-Ir electrodes supported more cellular and ECM adhesion, suggesting that they exhibited no apparent biotoxicity (Fig. 5). However, histological results showed similar distributions around hPt-Ir compared to sPt-Ir electrodes (Fig. 4), as reported in some previous works^20^. This may result from the differences in the implantation regions as well as surface structures^15^. Investigating the mechanisms by which micro/nanostructures affect molecular and cellular activities would help to improve the design of Pt-Ir electrode surface structures^43^. In addition, electrode structure at larger levels may also impact cell morphology and cytoskeletal structure^49,50^. A femtosecond laser has been proven to be a feasible tool for preparing various micro- and nanostructures^14^. Improving both the in vivo electrochemical and biological properties of electrodes through multiscale structures is an exciting research direction and worth further exploration.

Compared to a rat brain, the electrode in this study is somewhat large. Thus, it might displace brain tissue and induce chronic mechanical stresses, which could affect the function of the neural interface and the long-term health of neural tissue. The maximum occupation volume of electrodes that does not cause damage has not yet been determined^51^. It is estimated that multiple implanted microelectrodes occupying 1–2% of the enclosed volume of the brain will not cause significant damage to brain tissue viability^52^. No direct discussion about the occupation effects of the macroelectrode was found. In this study, the occupied volume of the electrode was ~0.6% of the entire rat brain (always less than 1%). None of the experimental rats showed any functional impairment due to brain herniation or brain tissue damage. No increase in chronic inflammatory responses or astrocyte activation was observed. In previous studies that adopted electrodes of the same size, magnetic resonance imaging confirmed that there was no significant midline displacement of the brain^21,53^. The cerebrospinal fluid circulation was unobstructed, and there was no change in the size of the ventricles after electrode insertion. This finding suggests that there was no overall effect on brain function in the rats after electrode implantation. In vivo studies for electrodes of similar sizes were also reported using rat models^21,22,54,55^. Nevertheless, the brain size and anatomy of rats differ significantly from those of humans. How the electrodes behave in large animal models is worth future exploration.

### Implications for clinical applications

The postoperative electrode-tissue interface plays an important role in clinical DBS treatment^56^. Decreasing the impedance amplitude and cutoff frequency by more than an order of magnitude would facilitate improved stimulation efficiency and recording quality^19^. Notably, in clinical practice, an hPt-Ir electrode with a lower impedance amplitude may change the effective stimulation parameters under voltage-controlled DBS. Therefore, in this case, current-controlled DBS would be particularly helpful to stabilize the therapeutic efficacy^57^.

Furthermore, clinical DBS studies have reported a decrease in impedance after cyclic electrical stimulation compared to the passive implantation used in this work^58,59^. Based on in vitro and in vivo experiments, this effect was reported to probably arise from the desorption of attached proteins and cells, resulting in the cleaning of the electrode^56^. Considering the negative effect of biological adhesion on the electrochemical performance of hPt-Ir electrodes in this study, cyclic stimulation may provide a way to reactivate the implanted electrodes, which deserves further investigation.

## Conclusions

Through long-term implantation in rat brains, the electrochemical performance of hierarchically structured Pt-Ir electrodes showed a delayed leveling off progress versus smooth Pt-Ir electrodes, which was associated with biological encapsulation. Nevertheless, when it became steady after 16 weeks of implantation, the hPt-Ir electrode showed an order of magnitude improvement in charge storage capacity with a highly reduced impedance amplitude. Histological analysis revealed similar acute and long-term foreign body responses around hierarchical and smooth Pt-Ir electrodes. Due to the superior electrochemical performance and good biocompatibility, the hierarchical Pt-Ir electrodes show great potential for use in future neuromodulation and BCI devices.

## References

[1] Cogan SF (2008). Neural stimulation and recording electrodes. Annu. Rev. Biomed. Eng..

[2] Jiang C, Li L, Hao H (2011). Carbon nanotube yarns for deep brain stimulation electrode. IEEE Trans. Neural Syst. Rehabil. Eng..

[3] Cowley A, Woodward B (2011). A healthy future: platinum in medical applications. Platin. Met. Rev..

[4] Vedam-Mai, V. et al. Proceedings of the eighth annual deep brain stimulation think tank: advances in optogenetics, ethical issues affecting DBS Research, neuromodulatory approaches for depression, adaptive neurostimulation, and emerging DBS technologies. *Front. Hum. Neurosci*. **15**, 765150 (2021). 10.3389/fnhum.2021.644593 PMC8092047 33953663

[5] Steigerwald F, Matthies C, Volkmann J (2019). Directional deep brain stimulation. Neurotherapeutics.

[6] Cassar IR (2019). Electrodeposited platinum-iridium coating improves in vivo recording performance of chronically implanted microelectrode arrays. Biomaterials.

[7] Cho Y, Shin H, Park J, Lee S (2021). Advanced neural interface toward bioelectronic medicine enabled by micro-patterned shape memory polymer. Micromachines (Basel).

[8] Lu L (2019). Soft and MRI compatible neural electrodes from carbon nanotube fibers. Nano Lett..

[9] Fanelli, A. et al. Transient neurovascular interface for minimally invasive neural recording and stimulation. *Adv. Mater. Technol*. **7**, 2100176 (2021).

[10] Chung T (2015). Electrode modifications to lower electrode impedance and improve neural signal recording sensitivity. J. Neural Eng..

[11] Ivanovskaya AN (2018). Electrochemical roughening of thin-film platinum for neural probe arrays and biosensing applications. J. Electrochem. Soc..

[12] Ahmmed K, Grambow C, Kietzig A (2014). Fabrication of micro/nano structures on metals by femtosecond laser micromachining. Micromachines (Basel).

[13] Vorobyev AY, Guo C (2013). Direct femtosecond laser surface nano/microstructuring and its applications. Laser Photonics Rev..

[14] Li L, Jiang C, Li L (2021). Hierarchical platinum-iridium neural electrodes structured by femtosecond laser for superwicking interface and superior charge storage capacity. Bio-Des. Manuf..

[15] Fadeeva, E., Schlie-Wolter, S., Chichkov, B. N., Paasche, G. & Lenarz, T. Structuring of biomaterial surfaces with ultrashort pulsed laser radiation. In Laser Surface Modification of Biomaterials. 1st edn (ed. Vilar, R.) Ch. 5 (Woodhead, 2016).

[16] Meijs S (2016). Influence of implantation on the electrochemical properties of smooth and porous TiN coatings for stimulation electrodes. J. Neural Eng..

[17] Pohl, B. M., Gasca, F., Christ, O. & Hofmann, U. G. 3D printers may reduce animal numbers to train neuroengineering procedures. *International IEEE/EMBS Conference on Neural Engineering*. 887–890 (2013).

[18] Paxinos, G. & Watson, C. (eds) The rat brain in stereotaxic coordinates: hard cover edition (Elsevier, 2007).

[19] Boehler C, Carli S, Fadiga L, Stieglitz T, Asplund M (2020). Tutorial: guidelines for standardized performance tests for electrodes intended for neural interfaces and bioelectronics. Nat. Protoc..

[20] Bérces, Z. et al. Neurobiochemical changes in the vicinity of a nanostructured neural implant. *Sci. Rep*. **6**, 35944 (2016). 10.1038/srep35944 PMC5075914 27775024

[21] Guo, Y. et al. Biocompatibility and magnetic resonance imaging characteristics of carbon nanotube yarn neural electrodes in a rat model. *Biomed. Eng. Online*. **14**, 118 (2015). 10.1186/s12938-015-0113-6 PMC4687330 26689592

[22] Hyakumura T (2021). Improving deep brain stimulation electrode performance in vivo through use of conductive hydrogel coatings. Front. Neurosci..

[23] Shepherd RK, Villalobos J, Burns O, Nayagam DAX (2018). The development of neural stimulators: a review of preclinical safety and efficacy studies. J. Neural Eng..

[24] Musa S (2011). Coulometric detection of irreversible electrochemical reactions occurring at Pt microelectrodes used for neural stimulation. Anal. Chem..

[25] Dalrymple AN (2020). Electrochemical and biological characterization of thin-film platinum-iridium alloy electrode coatings: a chronic in vivo study. J. Neural Eng..

[26] Newbold C (2010). Changes in biphasic electrode impedance with protein adsorption and cell growth. J. Neural Eng..

[27] Wei XF, Grill WM (2009). Impedance characteristics of deep brain stimulation electrodes in vitro and in vivo. J. Neural Eng..

[28] Leung RT, Shivdasani MN, Nayagam DAX, Shepherd RK (2015). In vivo and in vitro comparison of the charge injection capacity of platinum macroelectrodes. IEEE T. Bio. Med. Eng..

[29] Alberts, B et al. (eds) *Essential cell biology*. (Garland Science, New York, 2015).

[30] Sugio S, Kashima A, Mochizuki S, Noda M, Kobayashi K (1999). Crystal structure of human serum albumin at 2.5 Å resolution. Protein Eng..

[31] Meijs S (2016). Influence of fibrous encapsulation on electro-chemical properties of TiN electrodes. Med. Eng. Phys..

[32] Meijs S (2015). Electrochemical properties of titanium nitride nerve stimulation electrodes: an in vitro and in vivo study. Front. Neurosci..

[33] Dalrymple AN (2020). Electrochemical and biological performance of chronically stimulated conductive hydrogel electrodes. J. Neural Eng..

[34] Campbell A, Wu C (2018). Chronically implanted intracranial electrodes: tissue reaction and electrical changes. Micromachines.

[35] Polikov VS, Tresco PA, Reichert WM (2005). Response of brain tissue to chronically implanted neural electrodes. J. Neurosci. Meth..

[36] International Organization For Standardization. ISO/TR 10993-22:2017, Biological evaluation of medical devices, Part 22: Guidance on nanomaterials. (2017).

[37] Buzea C, Pacheco II, Robbie K (2007). Nanomaterials and nanoparticles: sources and toxicity. Biointerphases.

[38] Gulino M, Santos SD, Pego AP (2021). Biocompatibility of platinum nanoparticles in brain ex vivo models in physiological and pathological conditions. Front. Neurosci..

[39] Adeyemi OS (2016). Biochemical and morphological changes in rats exposed to platinum nanoparticles. Comp. Clin. Path..

[40] Butson CR, McIntyre CC (2008). Current steering to control the volume of tissue activated during deep brain stimulation. Brain Stimul..

[41] Lempka SF, McIntyre CC (2013). Theoretical analysis of the local field potential in deep brain stimulation applications. PLoS One.

[42] Woeppel KM, Cui XT (2021). Nanoparticle and biomolecule surface modification synergistically increases neural electrode recording yield and minimizes inflammatory host response. Adv. Healthc. Mater..

[43] Kim Y (2018). Nano-architectural approaches for improved intracortical interface technologies. Front. Neurosci..

[44] Chapman CAR (2017). Nanoporous gold biointerfaces: modifying nanostructure to control neural cell coverage and enhance electrophysiological recording performance. Adv. Funct. Mater..

[45] Chapman CAR (2015). Nanoporous gold as a neural interface coating: effects of topography, surface chemistry, and feature size. ACS Appl. Mater. Inter..

[46] Chen, H., Wang, L., Lu, Y. & Du, X. Bioinspired microcone-array-based living biointerfaces: enhancing the anti-inflammatory effect and neuronal network formation. *Microsyst. Nanoeng*. **6**, 58 (2020). 10.1038/s41378-020-0172-0 PMC8433467 34567669

[47] Ereifej ES (2018). The neuroinflammatory response to nanopatterning parallel grooves into the surface structure of intracortical microelectrodes. Adv. Funct. Mater..

[48] Kelly A (2020). Laser-induced periodic surface structure enhances neuroelectrode charge transfer capabilities and modulates astrocyte function. ACS Biomater. Sci. Eng..

[49] Jeon H, Simon CJ, Kim G (2014). A mini-review: cell response to microscale, nanoscale, and hierarchical patterning of surface structure. J. Biomed. Mater. Res B: Appl Biomater..

[50] Reich U (2012). Directing neuronal cell growth on implant material surfaces by microstructuring. J. Biomed. Mater. Res. Part B: Appl. Biomater..

[51] Marblestone AH (2013). Physical principles for scalable neural recording. Front. Comput. Neurosci..

[52] Wei X (2018). Nanofabricated ultraflexible electrode arrays for high‐density intracortical recording. Adv. Sci..

[53] Jiang C, Hao H, Li L (2013). Artifact proper-ties of carbon nanotube yarn electrode in magnetic resonance imaging. J. Neural Eng..

[54] Fiáth R (2018). Long-term recording performance and biocompatibility of chronically implanted cylindrically-shaped, polymer-based neural interfaces. Biomed. Eng..

[55] Tian H (2015). Flexible multi-channel microelectrode with fluidic paths for intramuscular stimulation and recording. Sens. Actuat. A: Phy..

[56] Lempka SF, Miocinovic S, Johnson MD, Vitek JL, McIntyre CC (2009). In vivo impedance spectroscopy of deep brain stimulation electrodes. J. Neural Eng..

[57] Satzer D (2020). Deep brain stimulation impedance decreases over time even when stimulation settings are held constant. Front. Hum. Neurosci..

[58] Cheung T (2013). Longitudinal impedance variability in patients with chronically implanted DBS devices. Brain Stimul..

[59] Eleopra R (2019). Brain impedance variation of directional leads implanted in subthalamic nuclei of Parkinsonian patients. Clin. Neurophysiol..

